# Gardenoside attenuates *Staphylococcus aureus*-induced mastitis by inhibiting inflammation and ferroptosis through Nrf2/SLC7A11/GPX4 signaling pathway

**DOI:** 10.1128/spectrum.02262-24

**Published:** 2024-11-29

**Authors:** Guoqing Sui, Wei Jiang, Lianyue Guan

**Affiliations:** 1 Department of Hepatobiliary-Pancreatic Surgery, China-Japan Union Hospital of Jilin University, Changchun, China; 2 Department of Ultrasound, China-Japan Union Hospital of Jilin University, Changchun, China; Nanjing Agricultural University, Nanjing, China

**Keywords:** mastitis, ferroptosis, Nrf2, inflammation, gardenoside

## Abstract

The occurrence of mastitis is mainly due to the infection of mammary tissue by pathogens, which causes the inflammation of mammary tissue. Gardenoside (GAD), an iridoid active ingredient extracted from *Gardenia Jasminoides Ellis*, has been revealed to exhibit anti-inflammatory and anti-oxidative roles. However, the therapeutic effect of GAD on mastitis remains unclear. Our aim was to identify the therapeutic effect of GAD on *Staphylococcus aureus (S. aureus*)-induced mastitis and clarify its mechanism. To carry out this work, *S. aureus*-induced mastitis of mice model was established. Enzyme-Linked Immunosorbent Assay (ELISA) was conducted to detect the production of Interleukin-1β (IL-1β) and tumor necrosis factor-α (TNF-α). The biochemical method was used to detect the antioxidant factors glutathione (GSH), malondialdehyde (MDA), and iron content. Western blot was used to detect the expression of Nrf2/SLC7A11/GPX4 signal-related proteins. The results demonstrated that GAD alleviated Myeloperoxidase (MPO) activity, IL-1β and TNF-α production, and NF-κB activation. MDA and iron contents were also inhibited by GAD. Meanwhile, GSH level and GPX4, SLC7A11, and ferritin expressions were increased by GAD treatment. In addition, the expressions of Nrf2 and HO-1 were upregulated by GAD. In conclusion, GAP may inhibit *S. aureus*-induced mastitis in mice by triggering the Nrf2/SLC7A11/GPX4 signaling pathway and alleviating inflammation and ferroptosis.

**IMPORTANCE:**

Mastitis, as an important disease that endangers the development of the dairy industry, causes huge economic losses to the breeding industry. *Staphylococcus aureus* is one of the important pathogenic bacteria that cause mastitis. Antibiotics are considered to be the first choice in the treatment of the *S. aureus*-induced mastitis. However, the overuse of antibiotics leads to bacterial resistance and antibiotic residues. Therefore, this study explored whether effective extracts of traditional herbs could be used as alternatives to antibiotics.

## INTRODUCTION

Mastitis is a relatively common disease in the process of dairy farming, which leads to breast somatic cell increase, edema and hyperplasia of breast interstitial, atrophy and degeneration of acinous cavity, etc., which seriously affects milk production and reduces milk quality (1). Moreover, residual bacteria and toxins pose a great threat to the health of consumers (2). The main causes of mastitis in dairy cows include breed, feeding management, and pathogenic microbial infection, among which pathogenic microbial infection is the main cause of mastitis (3). There are more than 150 types of bacteria that cause mastitis. Among them, *Staphylococcus aureus* (*S. aureus*) is the most common and important pathogen causing mastitis in many countries, with a prevalence of 5%–50%, which causes huge economic losses (4). Antibiotics are considered to be the first choice for the treatment of the *S. aureus*-induced mastitis (5). However, the overuse of antibiotics leads to bacterial resistance and antibiotic residues. Therefore, this study explored whether effective extracts of traditional herbs could be used as alternatives to antibiotics.

GAD is an iridoid active ingredient extracted and purified from *Gardenia Jasminoides Ellis*. It has been known to exhibit anti-oxidative and anti-inflammatory effects (6). A previous study exhibited that GAD mitigated inflammation and inhibited Extracellular matrix (ECM) degradation in IL-1β-treated rat chondrocytes by suppressing NF-κB activity (7). It has been reported that GAD has a protective effect on free fatty acid (FFA)-induced cellular steatosis in HepG2 cells by suppressing supernatant inflammatory cytokine production and intracellular NF-κB signaling pathway (8). Meanwhile, GAD inhibits oxidative stress and inflammatory damage in non-alcoholic rats by inhibiting the NLRP3 signaling pathway (9). However, whether GAD possesses the effect to ameliorate *S. aureus*-induced mastitis and the underlying mechanism remains unclear.

In this study, a mice mastitis model induced by *S. aureus* was used to explore the protective effects of GAD on mastitis. The results showed that GAD can improve the pathological damage of mammary gland tissue, reduce the secretion of pro-inflammatory cytokines, and inhibit ferroptosis.

## RESULTS

### GAD alleviates *S. aureus*-induced mammary histological injury

**Fig 1:**
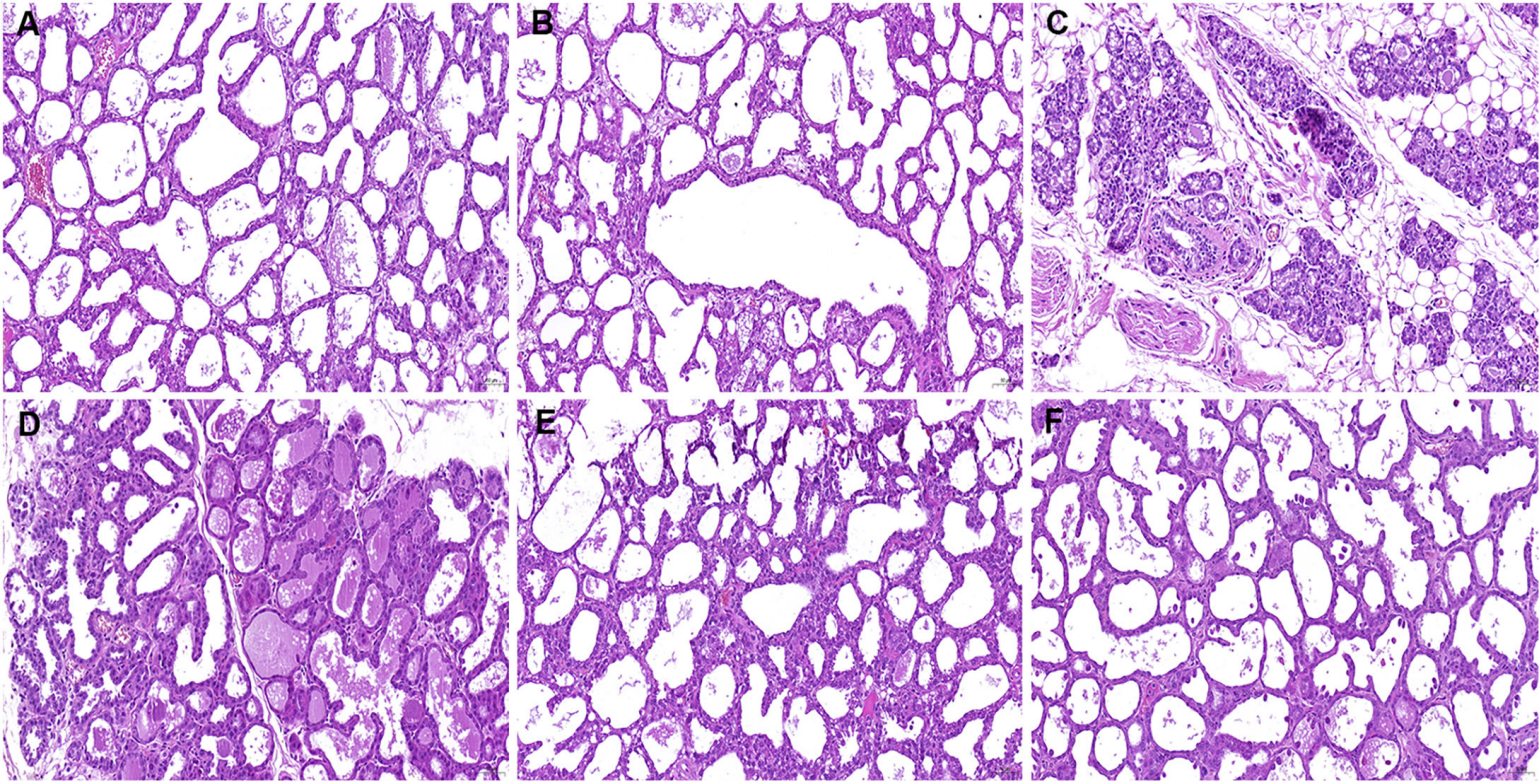
Effects of GAD on *S. aureus*-induced mammary histopathological changes. Histopathologic sections of mammary tissues (hematoxylin-eosin, H&E × 100). (**A**) Control, (**B**) GAD (40 mg/kg) group, (**C**) *S. aureus* group, and (**D-F**) GAD (10, 20, and 40 mg/kg) + *S. aureus* groups.

To investigate the therapeutic effects of GAD on *S. aureus*‐induced mastitis in mice, we conducted a dose‐dependent study using various doses of GAD (10, 20, and 40 mg/kg). The results showed that GAD treatment significantly alleviated *S. aureus*‐induced mammary damage, which was mainly manifested as inflammatory cell infiltration and structure destruction (Fig. 1).

### GAD alleviates *S. aureus*‐induced inflammatory response

**Fig 2:**
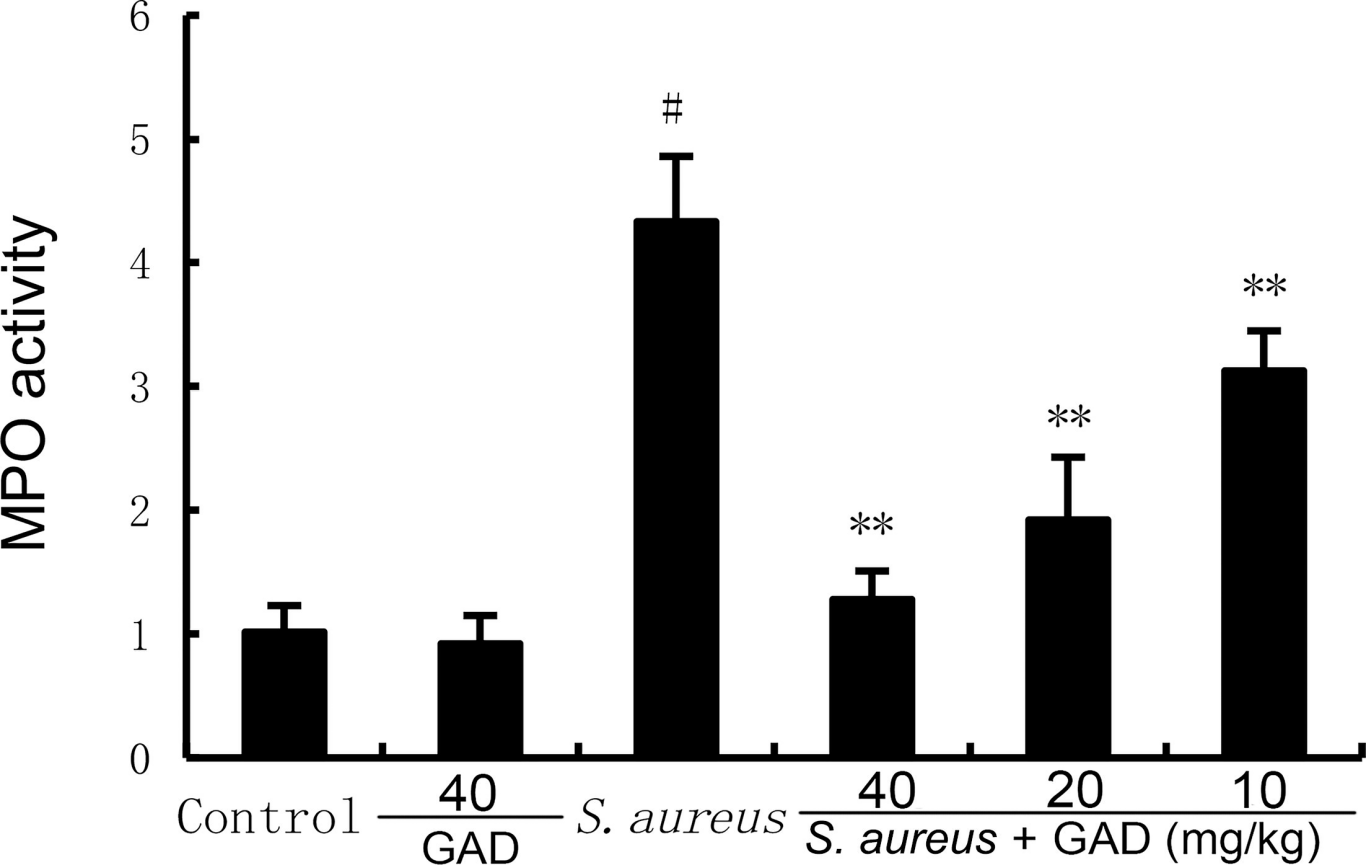
Effect of GAD on MPO activity in the mammary gland. The values presented are the mean ± SD. ^#^*P* < 0.01 is significantly different from the control group; ^**^P < 0.01 are significantly different from the *S. aureus* group.

**Fig 3:**
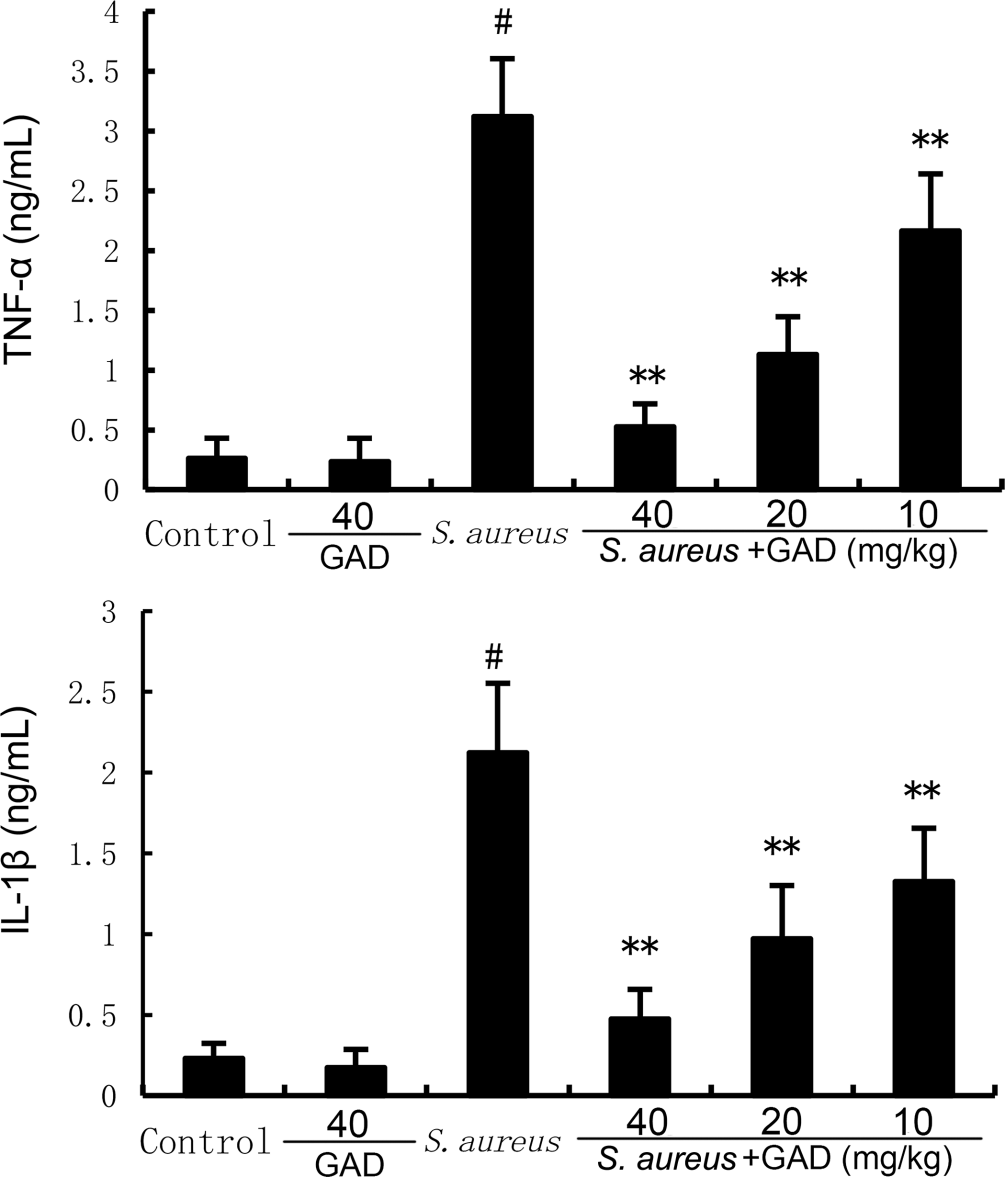
Effect of GAD on inflammatory cytokine production in the mammary gland. The values presented are the mean ± SD. ^#^*P* < 0.01 is significantly different from control group; ^**^P < 0.01 are significantly different from the *S. aureus* group.

MPO activity and inflammatory cytokine production were tested to assess mammary inflammatory levels. As demonstrated in Fig. 2 and 3, MPO activity, TNF‐α production, and IL‐1β production increased markedly in *S. aureus*‐treated mice. Likewise, GAD markedly reduced the elevated levels of inflammatory markers caused by *S. aureus*, including MPO (Fig. 2), TNF‐α, and IL‐1β (Fig. 3).

### GAD alleviates NF-κB activation in *S. aureus*-induced mastitis

**Fig 4:**
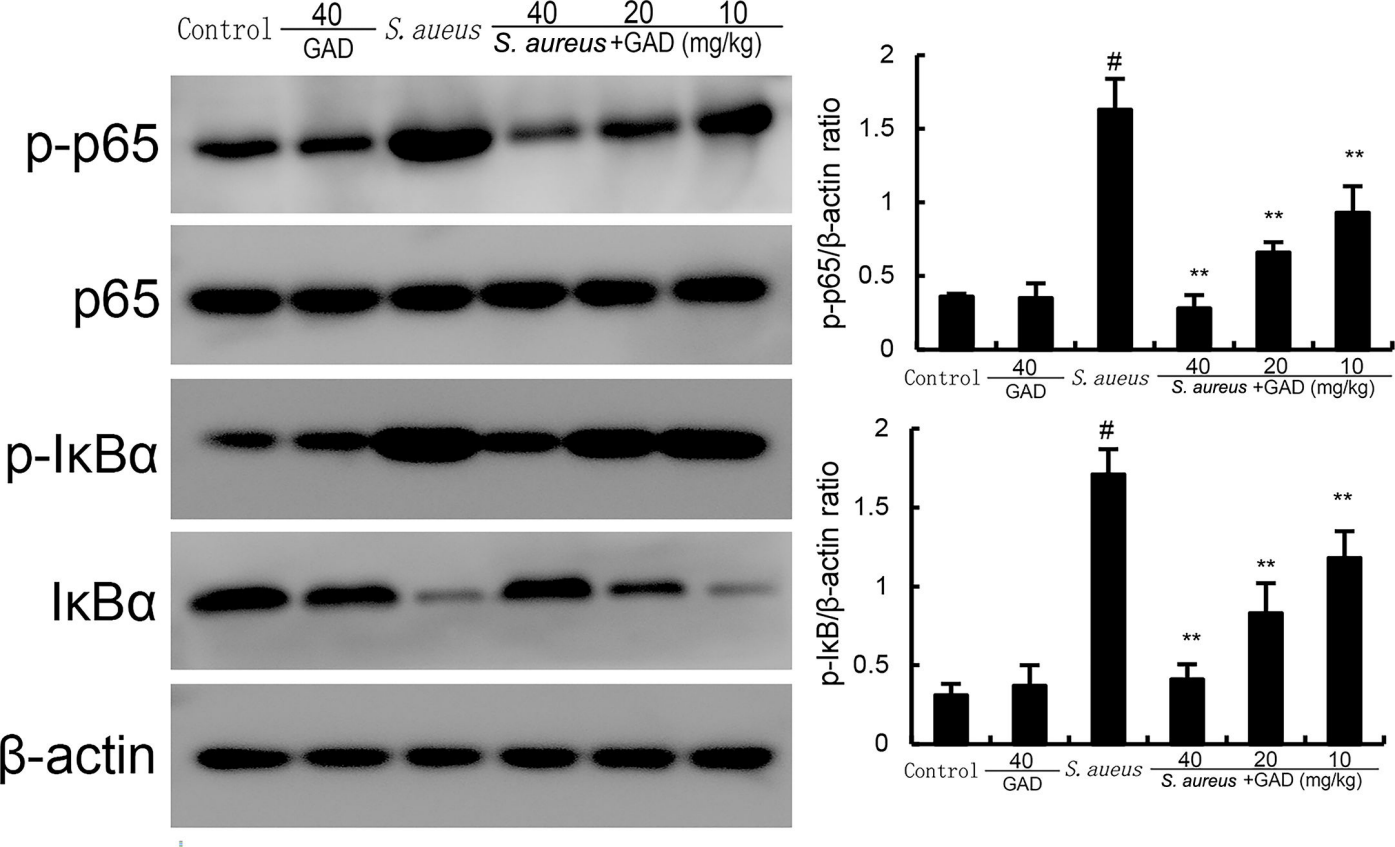
Effect of GAD on NF-κB activation in the mammary gland. The values presented are the mean ± SD. ^#^*P* < 0.01 is significantly different from the control group; ^**^P < 0.01 are significantly different from the *S. aureus* group.

To clarify the anti-inflammatory mechanism of GAD, NF-κB signaling pathway was measured. As illustrated in Fig. 4, phosphorylated IκB and NF-κB p65 expressions increased markedly in the *S. aureus* group. However, *S. aureus*-induced NF-κB activation was distinctly attenuated by GAD (Fig. 4).

### GAD alleviates *S. aureus*‐induced ferroptosis in mice

**Fig 5:**
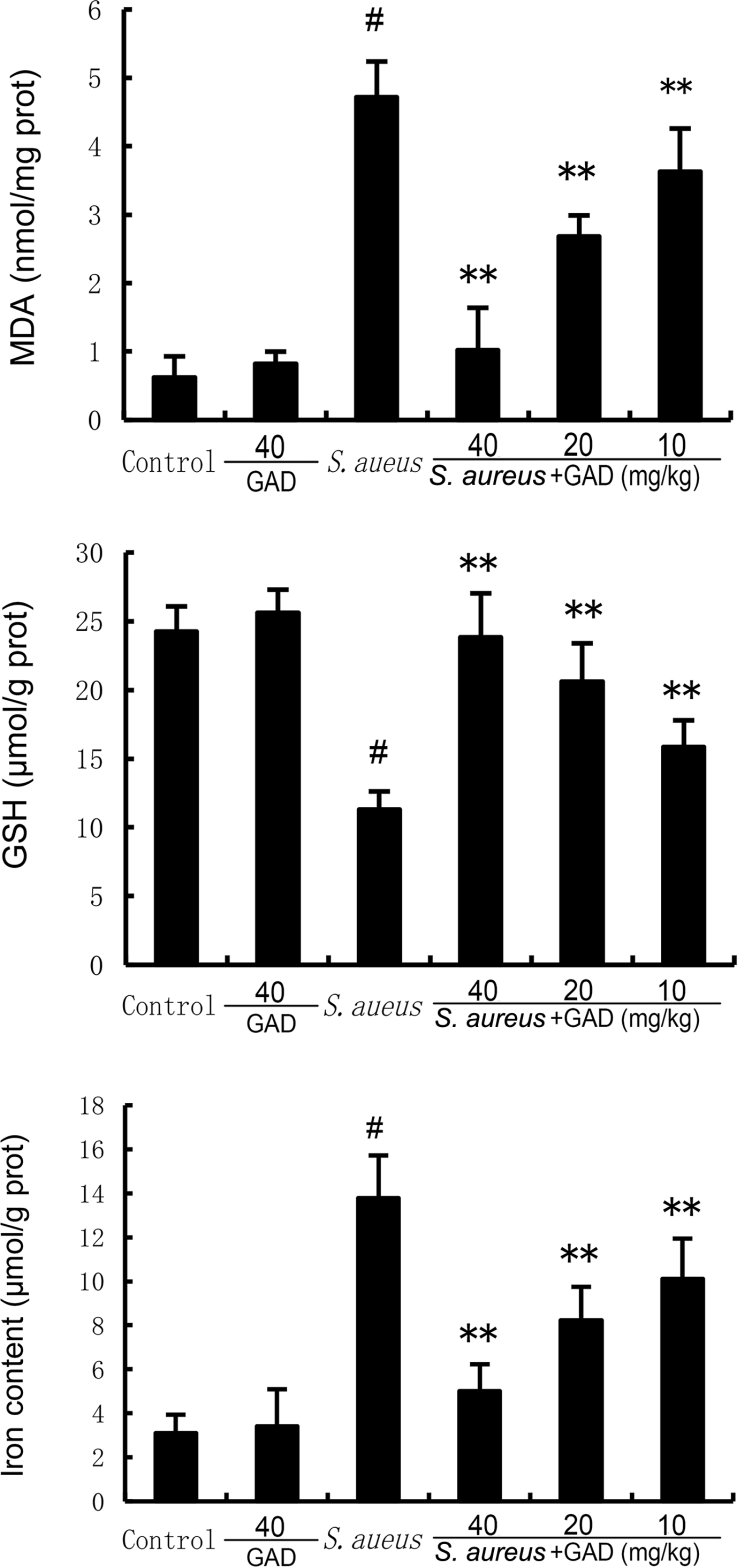
Effect of GAD on MDA, iron, and GSH production in the mammary gland. The values presented are the mean ± SD. ^#^*P* < 0.01 is significantly different from control group; ^**^P < 0.01 are significantly different from the *S. aureus* group.

**Fig 6:**
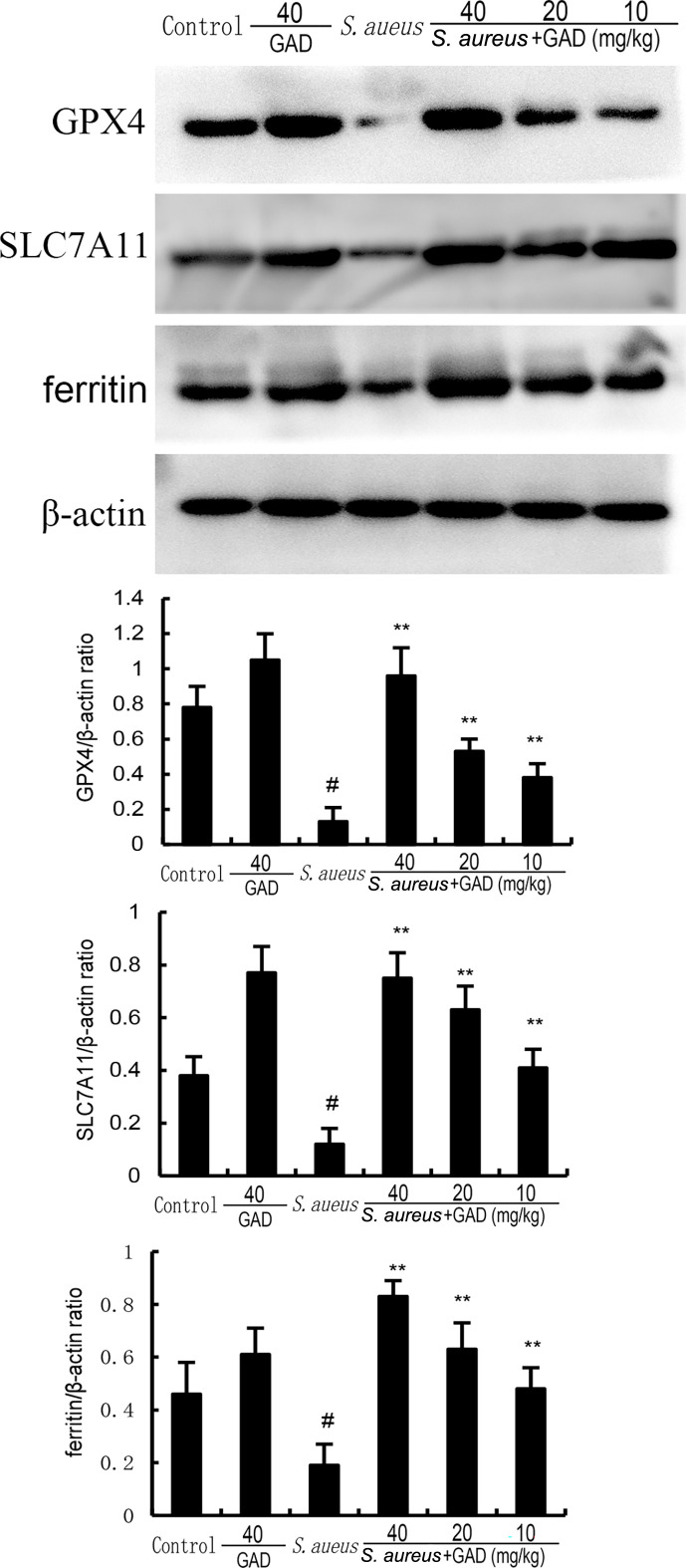
Effect of GAD on GPX4 and ferritin expression in the mammary gland. The values presented are the mean ± SD. ^#^*P* < 0.01 is significantly different from control group; ^**^P < 0.01 are significantly different from the *S.aureus* group.

To confirm the effects of GAD on ferroptosis, we examined the levels of MDA, GSH, iron, and the protein expressions of ferritin, SLC7A11, and GPX4. The results showed that S. aureus significantly decreased the levels of GSH, GPX4, ferritin, and SLC7A11 but increased the levels of MDA and iron concentration. However, these changes were prevented by the treatment of GAD (Fig. 5 and 6). These results suggested *S. aureus* could induce ferroptosis in mammary gland tissue. However, GAD markedly alleviated *S. aureu*s‐induced ferroptosis.

### Effects of GAD on Nrf2 and HO-1 expression

**Fig 7:**
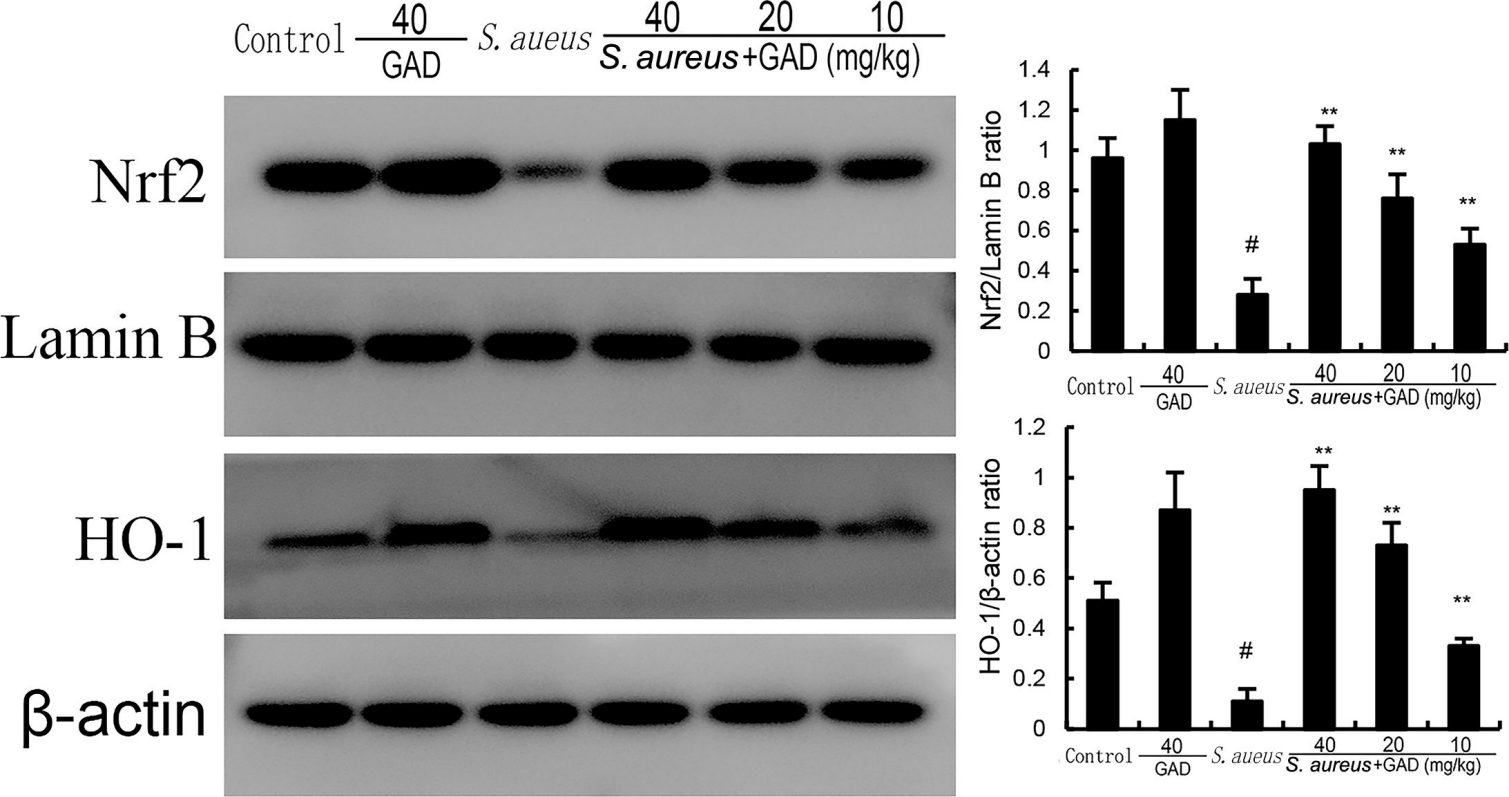
Effect of GAD on Nrf2 and HO-1 expression in the mammary gland. The values presented are the mean ± SD. ^#^*P* < 0.01 is significantly different from control group; ^**^P < 0.01 are significantly different from the *S.aureus* group.

Nrf2 was involved in inflammation and ferroptosis. In this study, the expression of Nrf2 and HO-1 was decreased by *S. aureus*. Treatment of GAD increased the expression of Nrf2 and HO-1 in a dose‐dependent manner (Fig. 7). These data suggest that GAD inhibited *S. aureus*‐induced mastitis by activating Nrf2 signaling pathway.

## DISCUSSION

Mastitis is a common infectious disease, causing significant economic losses in the dairy industry and affecting the quality and safety of dairy products. *S. aureus* is one of the major causative agents of mastitis in dairy cows. It has been shown that ferroptosis is involved in the pathological process of *S. aureus*‐induced mastitis (10). However, the role of GAD in ferroptosis and *S. aureus*‐associated mastitis and the underlying mechanisms are still unknown. In this study, we found that GAD could alleviate *S. aureus*‐induced mastitis in mice. These findings suggest that GAD could be a potential therapeutic agent for the treatment of *S. aureus*‐induced mastitis.

Inflammation is the host’s protective reaction to tissue dysfunction (11). Pro-inflammatory cytokines including TNF‐α and IL‐1β are closely related to the development and progression of mastitis (12). IL‐1β is produced in the early stages of infection and is thought to be an important mediator of inflammation (13). TNF‐α is a pluripotent and proinflammatory cytokine produced by activated macrophages during infection, thereby increasing leukocyte accumulation and amplifying the inflammatory cascade (14). These cytokines can also induce overactivation of neutrophils in tissues, which in turn exacerbates the disease process in the host (15). The data herein showed that TNF‐α, IL‐1β, neutrophil infiltration, and pathological damage were increased in the mammary gland. Nevertheless, GAD obviously decreased the level of inflammatory markers, including TNF‐α, IL‐1β, and MPO activity, and alleviated mammary pathological damage.

Ferroptosis is a cell damage mode closely related to changes in iron content, specifically an iron-dependent cell death (16). It has been confirmed that ferroptosis is closely related to the occurrence of mastitis, which may become a potential target for the prevention and treatment of mastitis (17). A previous study demonstrated that ferroptosis occurred in *S. aureus*-induced mastitis (18). Meanwhile, studies showed that inhibition of ferroptosis could protect mice against mastitis (19). The key mechanism of ferroptosis is the inhibition of GPX4 and the accumulation of lipid ROS in cells, resulting in the inactivation of cellular GSH (20). The cell surface cysteine-glutamate antiporter (system Xc−) is essential to the inhibition of ferroptosis. System Xc− takes up cystine, which is later reduced to cysteine and participates in the synthesis of GSH and excretes GSH. If the activity of system Xc− is inhibited, downstream GSH synthesis will decrease, which in turn leads to GPX4 inactivation (21). As far as is known, GPX4 is the only enzyme in the body that can reduce phospholipid hydroperoxide. Inactivation of GPX4 causes phospholipid hydroperoxide to accumulate, which eventually leads to ferroptosis (20). Erastin induces ferroptosis in multiple ways, one of which is via the inhibition of system Xc− activity (system Xc− is usually positively correlated with the expression of the light chain encoded by SLC7A11), resulting in a decrease in GSH expression, which eventually leads to ferroptosis (22). The antioxidant regulator Nrf2 is involved in the defense against oxidative stress (23). Under physiological conditions, Nrf2 combines with Keap1 in the cytoplasm. As soon as Nrf2 is stimulated, Nrf2 transfers into the nucleus to regulate the transcription of various genes, including SLC7A11 (24). Our results showed that GAD markedly inhibited *S. aureus*‐induced ferroptosis and activated the Nrf2/SLC7A11/GPX4 signaling pathway.

In conclusion, the data exhibited that GAD could inhibit *S. aureus*‐induced mastitis by attenuating inflammation and ferroptosis. The mechanism was through activating the Nrf2/SLC7A11/GPX4 signaling pathway.

## MATERIALS AND METHODS

### Reagents

TNF-α and IL-1β ELISA kits were purchased from Biolegend (San Diego, CA, USA). The antibodies were purchased from Affinity Biosciences. GAD (purity >99%) was purchased from MedChem Express. MPO, GSH, and MDA kits were purchased from the Nanjing Jiancheng Institute of Biological Engineering (Nanjing, China). The iron content kit was purchased from Solaibao Technology (Beijing, China).

### Animals and treatment

Bagg albino (BABL/C） mice (23–25 g, 72 females and 24 males) were purchased from Liaoning Changsheng Biotechnology Co., Ltd. (Benxi, Liaoning, China). All mice had free access to water and food. Female mice were housed with male mice in a 3:1 ratio until pregnancy, after which the male mice were removed. Seventy-two mice were divided into six groups: control group, GAD (40 mg/kg) group, *S. aureus* group, and *S. aureus* + GAD (10, 20, and 40 mg/kg) groups. The mammary glands of the fourth pair of mice were perfused with *S. aureus* [1 × 10^6^ Colony-Forming Units (CFU)] to induce mastitis (25). After 24 h, the mice were sacrificed, and mammary glands were collected for subsequent experiments. The animal experiments were carried out and approved by the Animal Administration Committee of Jilin University.

### Ferroptosis detection

GSH, MDA, and iron concentrations in mammary gland tissues were measured using the detection kits, respectively, according to the manufacturer’s instructions. The expressions of GPX4, ferritin, and SLC7A11 expression were detected using western blot analysis.

### H&E staining

Each group of mammary tissue was fixed with 4% paraformaldehyde for 48 h and cut into paraffin-embedded sections. Sections were stained with H&E, whereas pathological changes such as structural changes in breast tissue and inflammatory cell infiltration were observed later under light microscopy.

### MPO assay

Mammary tissue was collected and a 10% tissue homogenate was prepared. Then, the MPO level in mammary tissue was assayed using the MPO kit following the manufacturer’s instructions.

### ELISA assay

In total, 0.03 g of mammary gland tissue was collected and mixed with cold Phosphate Buffered Saline (PBS) at a ratio of 1:9, and the homogenate was prepared by tissue homogenizer. The homogenate was centrifuged for 10 min at 13,400 × *g* and 4°C, and the supernatant was collected to determine the concentrations of TNF-α and IL-1β following the manufacturer’s instructions.

### Western blot analysis

Total mammary tissue proteins were extracted using the Tissue Protein Extraction Kit (Thermo, USA), and protein concentrations were later measured using the BCA kit (Thermo, USA). Equal amounts (50 µg) of protein were transferred to Polyvinylidene Fluoride (PVDF) membranes after treatment with 10% or 12% sodium dodecyl sulfate-polyacrylamide gel electrophoresis (SDS-PAGE). PVDF membranes are closed at room temperature in 5% skim milk for 3 h and then incubated overnight at 4°C with a 1,000-fold dilution of the specific antibody. The next day, PVDF membranes were rinsed three times with Tris-Buffered Saline and Tween (TBST) for 20 min each time, and then incubated with goat anti-rabbit secondary antibody (1:10,000) or goat anti-mouse secondary antibody (1:10,000) for 2 h at room temperature. Finally, the signals on the PVDF membranes were examined using the ECL detection system.

### Statistical analysis

Experimental data were analyzed using GraphPad Prism 8.0 (Manufacturer, La Jolla, CA, USA) data software. One-way Analysis of Variance (ANOVA) (Dunnett’s *t*-test) and two-tailed Student’s *t*-test were used to analyze the means of normally distributed data differences. When *P* < 0.05 or *P* < 0.01, the results were considered statistically significant.

## Data Availability

The data that support the findings of this study are available from the corresponding author upon reasonable request.
